# Three-dimensional evaluation of a virtual setup considering the roots and alveolar bone in molar distalization cases

**DOI:** 10.1038/s41598-023-41480-z

**Published:** 2023-09-11

**Authors:** Jaewook Huh, Jing Liu, Jae-Hun Yu, Yoon Jeong Choi, Hee-Kap Ahn, Chooryung J. Chung, Jung-Yul Cha, Kyung-Ho Kim

**Affiliations:** 1 Department of Orthodontics, Yonsei University College of Dentistry, Seoul, Korea; 2 Department of Orthodontics, Institute of Craniofacial Deformity, Yonsei University College of Dentistry, Seoul, Korea; 3 Graduate School of Artificial Intelligence, Department of Computer Science and Engineering, Pohang University of Science and Technology, Pohang, Korea; 4 Institute for Convergence Research and Education in Advanced Technology, Yonsei University, Seoul, Korea; 5 Department of Orthodontics, Institute of Craniofacial Deformity, Gangnam Severance Dental Hospital, College of Dentistry, Yonsei University, Seoul, Korea; 6 Department of Orthodontics, Institute of Craniofacial Deformity, College of Dentistry, Institute for Innovation in Digital Healthcare, Yonsei University, 50-1 Yonsei-ro, Seodaemun-gu, Seoul, 03722 Korea

**Keywords:** 3-D reconstruction, Three-dimensional imaging, Occlusion, Orthodontics

## Abstract

We aimed to evaluate root parallelism and the dehiscence or fenestrations of virtual teeth setup using roots isolated from cone beam computed tomography (CBCT) images. Sixteen patients undergoing non-extraction orthodontic treatment with molar distalization were selected. Composite teeth were created by merging CBCT-isolated roots with intraoral scan-derived crowns. Three setups were performed sequentially: crown setup considering only the crowns, root setup-1 considering root alignment, and root setup-2 considering the roots and surrounding alveolar bone. We evaluated the parallelism and exposure of the roots and compared the American Board of Orthodontics Objective Grading System (ABO-OGS) scores using three-dimensionally printed models among the setups. The mean angulation differences between adjacent teeth in root setups-1 and -2 were significantly smaller than in the crown setup, except for some posterior teeth (*p* < 0.05). The amount of root exposure was significantly smaller in root setup-2 compared to crown setup and root setup-1 except when the mean exposure was less than 0.6 mm (*p* < 0.05). There was no significant difference in ABO-OGS scores among the setups. Thus, virtual setup considering the roots and alveolar bone can improve root parallelism and reduce the risk of root exposure without compromising occlusion quality.

Many studies have investigated the optimal alignment of teeth and occlusal relationships to achieve the best possible orthodontic treatment results^1,2^. Several methods for objectively evaluating orthodontic treatment outcomes have been proposed, including the American Board of Orthodontics Objective Grading System (ABO-OGS) and Peer Assessment Rating index^3^. However, to evaluate tooth alignment, previous studies have predominantly focused on clinical crown arrangement, and root alignment was not considered, aside from root parallelism evaluation using panoramic radiographs^1,4^. Appropriate root alignment is necessary to prevent iatrogenic dehiscence or fenestration^5^. However, panoramic radiographs have limited accuracy in assessing the relationship between the roots and surrounding alveolar bone^6,7^.

Virtual technology has been widely used in treatment planning as a means of simulating or predicting orthodontic treatment plans and outcomes^8^. Moreover, the virtual diagnostic setup has demonstrated similar reliability and accuracy as the manual setup, justifying its use in orthodontic diagnosis^9–12^. However, most of the current virtual setups only consider crowns, and limited research has been conducted on root arrangements and their relationship with the alveolar bone^13^. A recently proposed technique describes superimposing CBCT teeth images onto intraoral scans allowing root alignment evaluation during orthodontic treatment without additional radiation exposure^13–16^. The assessment of the conventional virtual setup through this method showed that a setup using only crowns was insufficient for obtaining optimal root alignment^13,17^.

The development of temporary anchorage devices (TADs) has increased the range of tooth movement in orthodontic treatment, which has facilitated the distalization of molars, enabling non-extraction treatment in some significant crowding cases^18,19^. However, if the position to which we plan to move the tooth means that roots will exit the alveolar bone, complications including dehiscence or fenestration^20^, or root resorption^21^ may occur unless the alveolar bone remodeling is complete^22^. The tooth may also fail to reach the desired location. Despite the importance of root movement within the alveolar bone for stable treatment results, few studies have quantified expected root exposure during or after orthodontic treatment. There have been many studies on the anatomical structures that limit posterior tooth movement in the maxilla and mandible^23,24^. However, research on the extent and distribution of root exposure occurring during posterior tooth movement is scarce. The simulation of orthodontic treatment during which the teeth are moved within the confines of the alveolar bone can help minimize the potential side effects, including root exposure, gingival recession, and root resorption that may arise as a result of tooth movement exceeding the limits of the alveolar bone^25^. It would also be meaningful to investigate the qualitative impact on occlusion when modifying the conventional setup method to avoid root exposure.

In this study, we aimed: 1) to evaluate the virtual setups of non-extraction molar distalization cases with or without consideration of the roots and alveolar bone in terms of the root parallelism and the occurrence of root exposure and 2) to qualitatively evaluate the occlusion in each of the virtual setups.

The null hypotheses of this study were as follows: 1) There is no difference in root parallelism and root exposure among virtual setups that consider the roots and alveolar bone and those that do not. 2) There is no difference in ABO OGS scores among virtual setups.

## Results

The ICC for angulation of the teeth was 0.987 and displayed high intra-rater reliability. The paired t-test did not reveal differences in angular measurements between the left and right sides of all teeth in the initial scan and thus these were pooled for analyses.

### Root parallelism between adjacent teeth

Upon measuring the angulation differences between the adjacent maxillary teeth, we observed that the long axes of the teeth in the root setup-1 were significantly more parallel (*p* < 0.05) than those in the crown setup. Specifically, this was observed in between the central and lateral incisors, lateral incisor and canine, and first and second premolars. In the mandible, the root setup-1 showed significantly greater parallelism (*p* < 0.05) between adjacent teeth in all areas, except between the second premolar and first molar, and the first and second molars (Table 1).

**Table 1:** Comparison of the mean differences of angulation between adjacent teeth for the three setup models (in Degrees).

	Location	Crown setup		Root setup-1		Root setup-2		*p-*value	Post-hoc
		Mean	SD	Mean	SD	Mean	SD		
Maxillary	1–2	7.35	4.79	5.92	4.39	5.89	4.59	0.014*	a > b
	2–3	5.82	5.00	4.13	3.53	4.22	3.12	0.008*	a > b,c
	3–4	5.27	3.40	4.63	2.24	4.80	2.14	0.380	NA
	4–5	5.68	4.58	4.01	2.37	3.98	2.15	0.007*	a > b,c
	5–6	3.51	2.97	2.52	1.54	2.64	1.74	0.088	NA
	6–7	2.58	2.30	2.41	2.19	2.49	2.40	0.666	NA
Mandibular	1–2	4.07	2.54	3.06	2.02	2.80	1.98	< 0.001*	a > b,c
	2–3	6.93	3.62	4.82	2.46	4.88	2.78	0.001*	a > b,c
	3–4	3.88	3.05	2.39	1.64	2.26	1.64	0.012*	a > b,c
	4–5	3.30	2.34	2.22	1.62	1.93	1.37	0.001*	a > b,c
	5–6	4.78	3.11	4.16	2.85	4.08	2.72	0.024*	NA
	6–7	4.22	2.96	4.24	3.12	4.38	3.28	0.723	NA

*p*-values were derived from repeated-measures ANOVA, *p < 0.05.

Bonferroni adjustment was used for multiple comparisons.

NA = not applicable.  a, Crown setup; b, Root setup-1; c, Root setup-2.

1–2, Between the central and lateral incisors; 2–3, Between the lateral incisor and canine; 3–4 Between the canine and first premolar; 4–5, Between the first and second premolars; 5–6, Between the second premolar and first molar; 6–7, Between the first and second molars.

### Root exposure measurements

Among the three setups, comparison of the amount of root exposure in the maxilla showed significant differences (*p* < 0.01) for all tooth types on the buccal side and incisors on the palatal side. In the mandible, we observed significant differences on the buccal side only in the anterior teeth (*p* < 0.001). On lingual side, all tooth types had significant differences (*p* < 0.01). The post-hoc tests demonstrated significantly less root exposure in root setup-2 compared to both crown setup and root setup-1 for all tooth types that displayed significant differences among setups except the mandibular canines on the buccal side and premolars on the lingual side. In contrast, root setup-1 did not show significant differences compared to the crown setup except in the maxillary canines on the buccal side and mandibular incisors on the lingual side (Table 2).

**Table 2:** Comparison of mean values of root exposure on buccal and palatal/lingual sides for setup models.

	Tooth type	Buccal (mm)								Palatal/lingual (mm)							
		Crown setup		Root setup-1		Root setup-2		*p-*value	Post-hoc	Crown setup		Root setup-1		Root setup-2		*p-*value	Post-hoc
		Mean	SD	Mean	SD	Mean	SD			Mean	SD	Mean	SD	Mean	SD		
Maxillary	Incisor	1.27	3.43	1.23	3.18	0.00	0.00	0.002*	a > c b > c	0.68	1.77	0.65	1.65	0.04	0.19	< 0.001*	a > c b > c
	Canine	2.70	4.75	1.96	4.29	0.00	0.00	< 0.001*	a > b a > c b > c	0.31	0.79	0.37	0.83	0.07	0.21	0.255	NA
	Premolar	1.01	2.84	0.91	2.69	0.00	0.00	0.002*	a > c b > c	0.00	0.00	0.00	0.00	0.00	0.00	NA	NA
	Molar	1.92	3.24	1.85	3.20	0.06	0.28	< 0.001*	a > c b > c	0.00	0.00	0.00	0.00	0.00	0.00	NA	NA
Mandibular	Incisor	1.20	2.31	1.24	2.29	0.05	0.37	< 0.001*	a > c b > c	1.27	2.07	1.12	1.98	0.04	0.18	< 0.001*	a > b a > c b > c
	Canine	0.55	1.65	0.55	1.66	0.01	0.06	0.018*	NA	1.04	1.53	1.08	1.75	0.17	0.57	0.002*	a > c b > c
	Premolar	0.02	0.16	0.02	0.17	0.04	0.34	0.867	NA	0.35	1.31	0.09	0.53	0.02	0.12	0.031*	NA
	Molar	0.03	0.23	0.00	0.00	0.04	0.34	0.607	NA	1.00	2.81	0.94	2.79	0.00	0.00	0.001*	a > c b > c

*p*-values were derived from the Friedman test, *p < 0.05.

Post-hoc tests were performed with the Wilcoxon signed rank test.

Bonferroni adjustment was used for multiple comparisons.

NA = not applicable.  a, Crown setup; b, Root setup-1; c, Root setup-2.

If a root extended beyond the trimmed border of alveolar bone, it was considered exposed.

### Comparison of the frequency of root exposure

The McNemar test revealed significant differences in the frequency of root exposure greater than 2 mm between the buccal and palatal/lingual sides on several locations (*p* < 0.05). In the crown setup and root setup-1, the maxillary posterior teeth displayed frequent root exposure on the buccal side whereas lingual root exposure was more frequent in the molars of the mandible (Table 3). In the crown setup, the maxillary canines showed significantly more frequent root exposure on the palatal side.

**Table 3:** Comparison of the frequency of root exposure greater than 2 mm on the buccal and palatal/lingual sides for the three setup methods.

		Maxilla							Mandible						
		Buccal		Palatal		no exposure		*p-*value	Buccal		Lingual		no exposure		*p-*value
Crown setup	Incisor	8	(12.5%)	7	(10.9%)	49	(76.6%)	1.000	14	(21.9%)	16	(25.0%)	34	(53.1%)	0.855
	Canine	9	(28.1%)	1	(3.1%)	22	(68.8%)	0.021	3	(9.4%)	7	(21.9%)	22	(68.8%)	0.344
	Premolar	8	(12.5%)	0	(0.0%)	56	(87.5%)	0.008	0	(0.0%)	5	(7.8%)	59	(92.2%)	0.063
	Molar	18	(28.1%)	0	(0.0%)	46	(71.9%)	< 0.001	0	(0.0%)	8	(12.5%)	56	(87.5%)	0.008*
Root setup-1	Incisor	9	(14.1%)	7	(10.9%)	48	(75.0%)	0.804	17	(26.6%)	13	(20.3%)	34	(53.1%)	0.584
	Canine	6	(18.8%)	1	(3.1%)	25	(78.1%)	0.125	3	(9.4%)	6	(18.8%)	23	(71.9%)	0.508
	Premolar	8	(12.5%)	0	(0.0%)	56	(87.5%)	0.008	0	(0.0%)	1	(1.6%)	63	(98.4%)	1.000
	Molar	18	(28.1%)	0	(0.0%)	46	(71.9%)	< 0.001	0	(0.0%)	8	(12.5%)	56	(87.5%)	0.008*
Root setup-2	Incisor	0	(0.0%)	0	(0.0%)	64	(100.0%)	NA	1	(1.6%)	0	(0.0%)	63	(98.4%)	1.000
	Canine	0	(0.0%)	0	(0.0%)	32	(100.0%)	NA	0	(0.0%)	1	(3.1%)	31	(96.9%)	1.000
	Premolar	0	(0.0%)	0	(0.0%)	64	(100.0%)	NA	1	(1.6%)	0	(0.0%)	63	(98.4%)	1.000
	Molar	1	(1.6%)	0	(0.0%)	63	(98.4%)	1.000	1	(1.6%)	0	(0.0%)	63	(98.4%)	1.000

*p*-values were derived from the McNemar test. *p < 0.05.

Data are presented as frequency (%).

### Evaluation of occlusion

The evaluation of the ABO-OGS scores did not reveal any significant differences among the setups in individual categories or in the overall score. The average overall scores for the crown setup, root setup-1, and root setup-2 were 13.31, 13.06, and 13.50, respectively (Table 4).

**Table 4:** Comparison of ABO-OGS score among the three setup models.

	Crown setup		Root setup-1		Root setup-2		*p-*value
	Mean	SD	Mean	SD	Mean	SD	
Alignment/rotation	1.56	1.75	1.50	1.63	1.38	1.63	0.094
Marginal ridges	2.75	2.29	2.75	2.41	2.94	2.38	0.395
Buccolingual inclination	1.50	1.26	1.50	1.21	1.94	1.39	0.111
Overjet	0.38	0.81	0.50	0.63	0.56	1.09	0.750
Occlusal contacts	4.31	2.70	4.19	2.48	4.25	2.52	0.916
Occlusal relationships	2.81	1.72	2.63	1.86	2.44	1.86	0.161
Total	13.31	4.14	13.06	4.43	13.50	4.93	0.760

*p*-values were derived from repeated-measures ANOVA.

## Discussion

The primary focus of orthodontic treatment has traditionally been on crown alignment, which has resulted in a limited number of studies on the three-dimensional (3-D) evaluation of the relationship between the roots^26^. The relationship between root proximity and periodontal problems^27^ and the effects of root parallelism on stability following orthodontic treatment has been studied^28–30^. However, the research on root alignment mainly relied on evaluating panoramic radiographs and has been challenging due to imaging artifacts and distortions^31^. Recently, methods for measuring the mesiodistal angulation and the faciolingual inclination of teeth, including roots, using CBCT have been proposed to overcome the limitations of panoramic radiographs^32–34^. In the present study, we measured angular values in 3-D and evaluated the parallelism of the teeth, including the roots. For the 3-D angular measurement of individual teeth, instead of projecting the long axes of all teeth onto a common coronal or sagittal plane, we set different reference planes for each tooth while keeping the same occlusal plane reference.

We observed that using a root setup significantly improved root parallelism. In comparison to crown setup, the root setups-1 and -2 had significantly smaller angular deviations between the adjacent teeth, except for a few posterior teeth. In contrast, we observed that no teeth had a significant difference between root setups-1 and -2, suggesting that the consideration of the alveolar bone did not impair root parallelism in the orthodontic setup. For aesthetic reasons, orthodontic setups are more often focused on improving parallelism in the anterior teeth rather than the posterior teeth, which was also reflected in the observations between setups performed in the present study.

As part of the study, inclination values of the roots were measured for the three setups. Among these measurements, the mandibular premolars exhibited the highest mean deviation of 2.6° between crown setup and root setup-2 (supplementary table S1). This may cause a change of less than 0.4 mm in the vertical height difference between buccal and palatal/lingual cusps in molars and premolars^35^. According to the ABO-OGS, a deviation of more than 1 mm is required to deduct points for the buccolingual inclination and occlusal contacts section of the scoring system^36^. Therefore, this degree of inclination change is not considered sufficient to cause a qualitative change in the occlusion.

Orthodontic tooth movement can be associated with the risk of root exposure in the case of reduced alveolar bone support^5,37^. Tooth movement without considering the morphology of the alveolar bone can increase the risk of dehiscence or fenestration^38^. Therefore, a setup that considers both the roots and alveolar bone can help to predict or prevent root exposure caused by orthodontic tooth movement. The reported frequency of the alveolar bone defect varies among studies^39,40^, and varied definitions of dehiscence exist^41–43^. Thus, quantitatively observing bone defects before and after orthodontic treatment has some limitations. Moreover, segmented root images do not accurately represent the actual roots owing to the minor gray scale difference between the roots and surrounding alveolar bone^44,45^. Additionally, there are several limitations in the accurate segmentation of alveolar bone. These include the relatively lower spatial resolution of CBCT compared to that of intraoral scanners^46^, the presence of artifacts generated during scanning^47^, and the challenges in determining the threshold settings in the volume rendering process^48^. Furthermore, alveolar bone remodeling by orthodontic tooth movement^22^ and the potential impacts of pathophysiological changes due to increasing age^49^ and periodontal disease^50^ were not considered in the present study and root exposure was evaluated assuming that the alveolar bone morphology would remain unchanged during orthodontic treatment. Hence, an observed root exposure in a virtual setup is less likely to represent dehiscence or fenestration accurately. Unfortunately, no method has been proposed to simulate alveolar bone remodelling in response to orthodontic tooth movement. Even in vivo studies of root exposure^51^ have not been able to take into account the various conditions such as age, gender, and general health. Nevertheless, we believe that the methodology’s efficacy remains apparent, as it could facilitate the reduction of root exposure without adversely affecting occlusal integrity or root parallelism, even under more stringent conditions where the beneficial impact of alveolar bone remodeling to mitigate root exposure was not present. To address the limitations in observing root exposure when comparing the frequency between buccal and palatal/lingual sides, we only considered teeth with root exposure greater than 2 mm^52^.

Limited research has been conducted on the root exposure that occurs during posterior molar movement on the buccal and palatal sides of the alveolar bone^51^. In this study, it was found that root exposure occurred on the buccal and palatal side in the crown setup simulating posterior tooth movement. The root setup-1, which did not consider the alveolar bone, did not significantly reduce root exposure except for maxillary canines and mandibular incisors. Only after considering the alveolar bone was a substantial reduction in root exposure achieved. We also observed that the location of frequent root exposure differed between the maxillary and mandibular dentitions and depended on the tooth type. A higher frequency of exposure on the palatal sides of the maxillary canines in the crown setup could be attributed to the posterior movement of the anterior teeth (1.82 ± 1.00 mm in the maxillary incisors and 0.85 ± 1.05 mm in the mandibular incisors; supplementary table S2). In contrast, the difference in exposure patterns in the posterior teeth of the maxillary and mandibular dentition was principally attributed to the morphological characteristics of the alveolar bone. In the maxilla, the buccal side of the tuberosity was identified as the posterior anatomical limitation for molar distalization due to the smaller distance from the distobuccal root to the cortex when compared to the distance from the palatal root to the cortex^23^. In contrast, the lingual cortex of the mandibular body represented the posterior anatomical constraint owing to the posteriorly diverging shape^24^.

In this study, we incorporated samples with skeletal Class I and mild-to-moderate Class II, III malocclusion. To ensure overall consistency in the study results, all samples exclusively consisted of non-extraction cases, with a common factor of posterior molar movement. Additionally, there was a consistent constraint placed on the anterior movement of incisors.

All setups achieved good-quality occlusion according to the ABO-OGS scores. Among the eight ABO-OGS categories, we observed no statistically significant difference among three setup groups, except for the evaluation of root parallelism using panoramic radiographs, which was not performed in the present study. Therefore, the superior occlusion quality of the crown setup did not deteriorate despite its additional modification to consider the roots and alveolar bone.

This study found that the virtual setup presented a potential risk of root exposure during molar distalization if the relationship between the roots and alveolar bone was not considered. However, developing a virtual setup with 3-D radiographic imaging for all patients is challenging because of the relatively high dose of radiation exposure in CBCT. Therefore, root setup should be considered carefully for treating patients who require substantial tooth movement that may exceed the boundary of the alveolar bone.

### Limitations of the study

Owing to the difficulty in achieving the ideal segmentation of the teeth and alveolar bone, we arbitrarily removed the portion of teeth above the alveolar crest and placed reference planes for dehiscence measurement. Follow-up studies should aim to accurately measure bone defects and reflect actual bone remodeling around the roots during orthodontic tooth movement. In addition, we only investigated the exposure pattern of the roots for the posterior movement of the dentition; thus, follow-up studies are warranted and should investigate transverse tooth movement.

## Conclusions

The virtual teeth setup using both crown and root composite data could improve root parallelism. Meanwhile, the virtual setup for molar distalization may demonstrate a potential risk of root exposure, which could be reduced through the virtual alignment of composite teeth within the alveolar bone. The prevalence of molar root exposure differed in the maxilla and the mandible, with higher incidences observed on the buccal side of the maxilla and the lingual side of the mandible. Moreover, the virtual setup considering both the roots and alveolar bone did not decrease the occlusion quality compared with that of the conventional crown setup. Therefore, the first null hypothesis was rejected and the second null hypothesis was not rejected.

## Methods

This study was approved by the Institutional Review Board of Yonsei University Dental Hospital (No. 2-2022-0070) and all research was carried out in accordance with the relevant guidelines and regulations. Informed consent was waived for this study by Institutional Review Board of Yonsei University Dental Hospital due to the retrospective design. All procedures in this study were conducted in accordance with the Declaration of Helsinki. The study sample was selected from 614 patients who visited the Department of Orthodontics, Yonsei University Dental Hospital, between February 2018 and February 2021, with treatment plans established by one of the authors (JY Cha). One-hundred-and-nine patients with non-extraction treatment plans underwent pre-treatment CBCT (Alphard VEGA; ASAHI Roentgen IND, Kyoto, Japan) and intraoral scans (Trios3; 3Shape, Copenhagen, Denmark). CBCT was set at 80 kV, 5.0 mA, 17 s scanning time, 154 mm × 154 mm field of view, and 0.3 mm voxel size (CBCT panoramic mode, low dose exposure). Of the 109 patients, 16 (5 male, 11 female; mean age 23.4 ± 8.2 years) were selected based on the following inclusion criteria. : (1) Skeletal Class I and mild-to-moderate Class II or Class III (ANB − 2.8° to 5.5°), (2) completely erupted permanent dentition, except for the third molars, and (3) Presence of crowding that is expected to be resolved by distalization of molars without forward movement of anterior teeth. The exclusion criteria were as follows: (1) developmental tooth anomalies, (2) cleft lip or palate, (3) severe periodontitis, (4) severe skeletal asymmetry, (5) anterior open bite, (6) history of orthodontic treatment with fixed appliances, (7) posterior crossbite, and (8) Less than 1 mm of combined posterior movement of the upper and lower first molars. CBCT was performed on the 109 non-extraction patients to investigate skeletal or periodontal problems, temporomandibular joint (TMJ) disorders, and impacted or missing teeth. However, among the patients who met the aforementioned criteria, few had reasons to undergo CBCT other than impacted third molars, minor skeletal asymmetry, or TMJ disorders, resulting in a small sample size of 16 patients (10 patients in Skeletal Class I, 2 in Class II, and 4 in Class III). Descriptive statistics of the enrolled patients are presented in supplementary table S3.

In previous studies comparing root angulation and inclination between expected root position using composite teeth and CBCT, a pilot study with five subjects demonstrated that the buccolingual inclination of maxillary canines required the largest sample size (30 teeth)^15,53^. Subsequently, a post hoc power analysis was conducted for the current study with 16 subjects (32 maxillary canines) using G*Power (version 3.1.9.7; Heinrich-Heine-Universität Düsseldorf, Düsseldorf, Germany), specifically for the inclination of maxillary canines. The partial eta squared and correlation coefficients among repeated measures were obtained from our preliminary study, which compared the inclination and angulation of each tooth type among the three setups using a sample of five subjects. For maxillary canines, the partial eta squared was determined to be 0.027, and the correlation coefficient 0.827. The effect size was calculated to be 0.11. In this study, the statistical power was estimated at 94% with a significance level of 0.05 for the repeated-measures analysis of variance (ANOVA) of maxillary canines.

### Segmentation of the maxilla, mandible, and individual teeth

**Figure 1:**
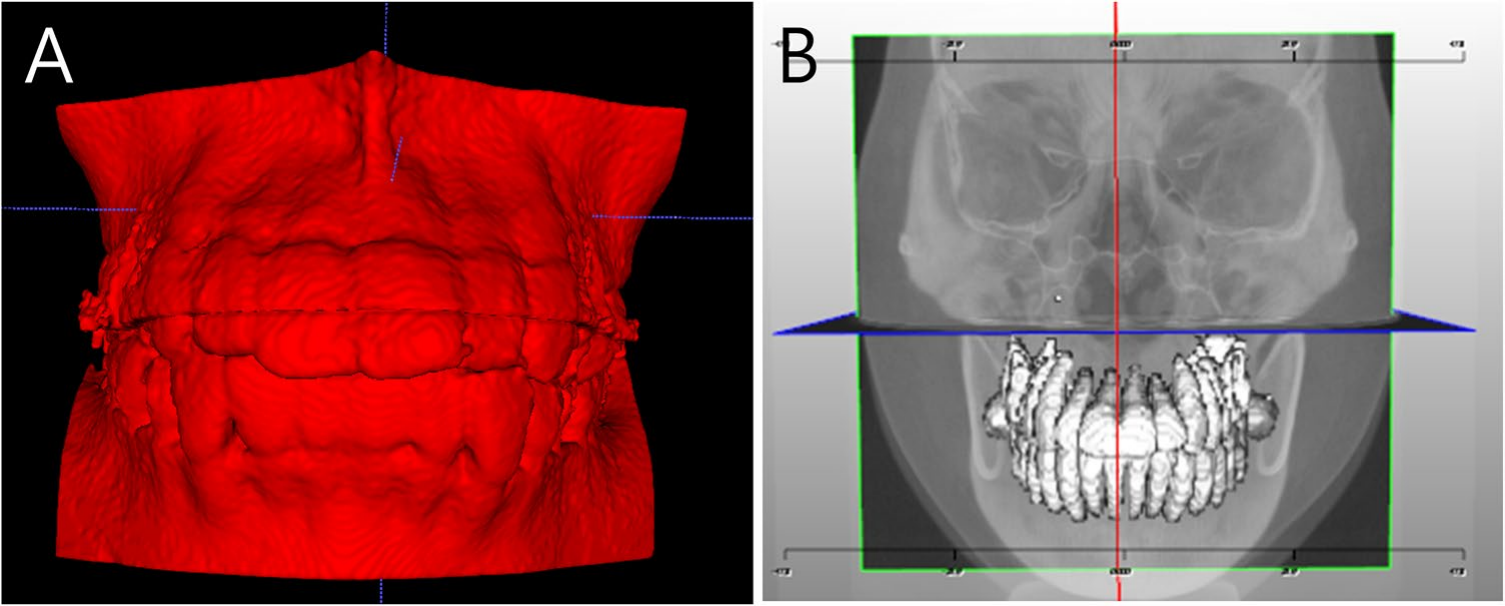
Segmentation of the maxilla, mandible, and teeth from CBCT. (**A**), segmentation of the maxilla and mandible. (**B**), segmentation of the individual teeth. CBCT, cone beam computed tomography.

Individual teeth were segmented from pre-treatment CBCT using LaonCBCT Viewer (version 1.2; Laon People; Gyeonggi-Do, Korea). The maxilla and mandible were separately segmented using the supervised classification mode of the ITK-SNAP program (version 3.8.0; http://www.itksnap.org), owing to the difference in gray scale^54^. Segmented images were saved as stereolithography (STL) files (Fig. 1).

### Preparation of the maxilla, mandible, and composite teeth

**Figure 2:**
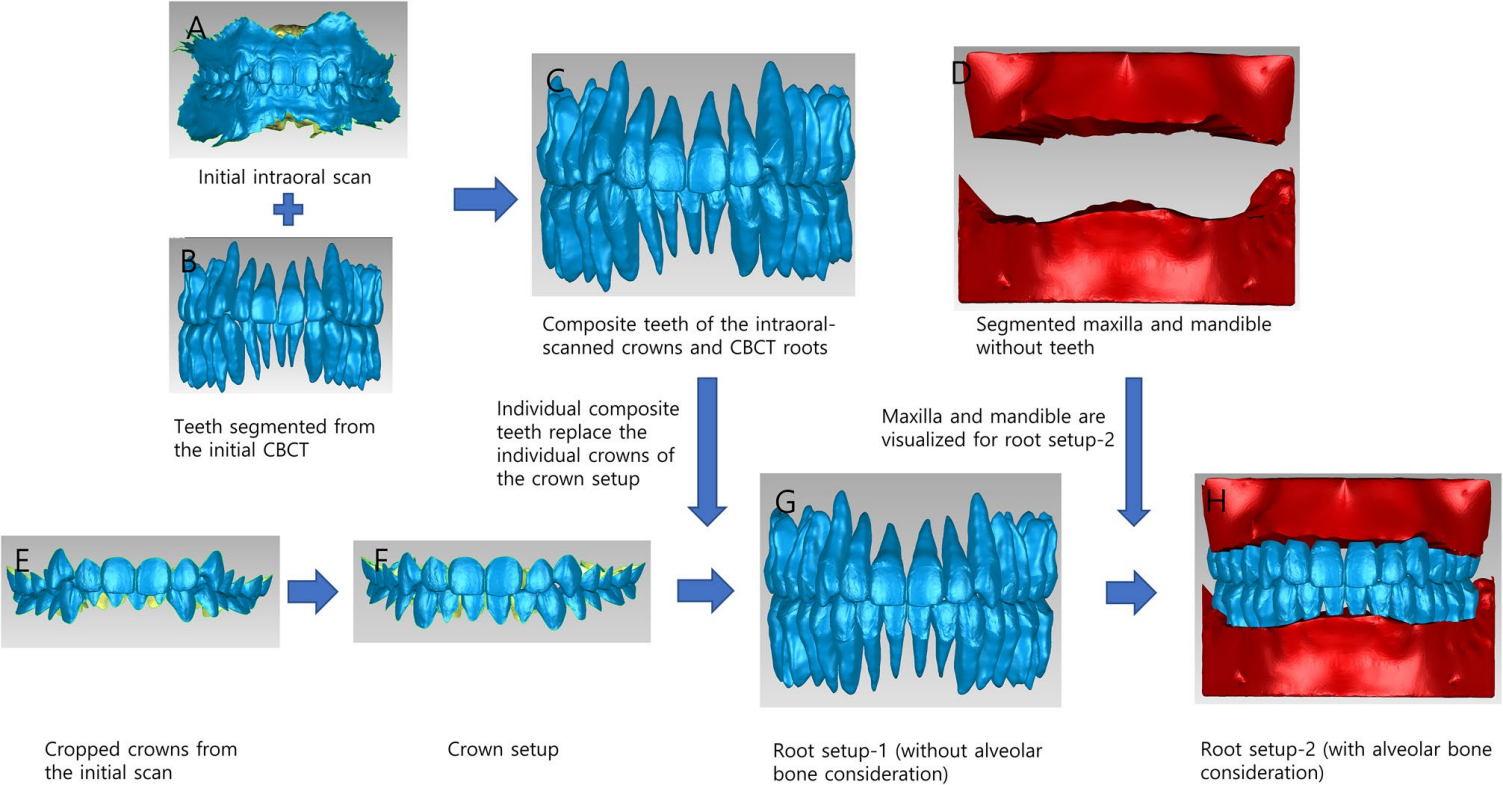
Setup process of the crown setup, root setup-1, and root setup-2. (**A**), The initial intraoral scan. (**B**), Teeth segmented from the initial CBCT. (**C**), Composite teeth of the intraoral-scanned crowns and CBCT roots. (**D**), Segmented maxilla and mandible with the tooth portion above the alveolar crest removed. (**E**), Crowns cropped from the initial scan. (**F**), Crown setup using the cropped crowns. (**G**), Root setup-1 using the composite teeth. (**H**), Root setup-2 considering the alveolar bone. CBCT, cone beam computed tomography.

The STL files of individually segmented teeth, including their root parts, were aligned with intraoral-scanned crowns to fabricate composite teeth. The maxillary and mandibular teeth were aligned in their position of maximum intercuspation. Subsequently, the crowns of the segmented teeth were removed and replaced by intraoral-scanned crowns. To establish a baseline for measuring root exposure from the alveolar bone, we trimmed the tooth portion above the alveolar crest from the maxilla and mandible STL files. The STL files were manipulated using Geomagic Control (version 2015; 3D Systems, Rock Hill, SC, USA) and Meshmixer (version 3.5.474; Autodesk, Mill Valley, CA, USA) (Fig. 2A–D). Meshmixer was used for trimming the teeth part from the alveolar bone and making the disc-shaped reference planes, and Geomagic Control was utilized otherwise.

### Virtual setup

The setups were constructed in three steps by a technician with 11 years of experience from Orapix (Seoul, Korea) using a virtual setup program (3Txer 2.0; Orapix, Seoul, Korea). The goals of all setups were set according to the actual treatment plan. First, a setup was fabricated using only intraoral-scanned crowns. The technician was instructed to relieve crowding with posterior molar movement and create a proper overjet/overbite relationship without anterior displacement of the incisors (crown setup). In the second step, the individual crowns of the crown setup were replaced with the composite teeth. Then, the setup was modified to ensure optimal inclination and angulation of the roots while maintaining the arch width and anterior–posterior position of the teeth (root setup-1). Finally, root setup-1 was refined to minimize dehiscence and fenestration in the presence of overlaying alveolar bone with the least possible deterioration in the root angulation, arch width and anterior–posterior relationship of the teeth (root setup-2) (Fig. 2E–H).

### Reference point assignments on the composite teeth

**Figure 3:**
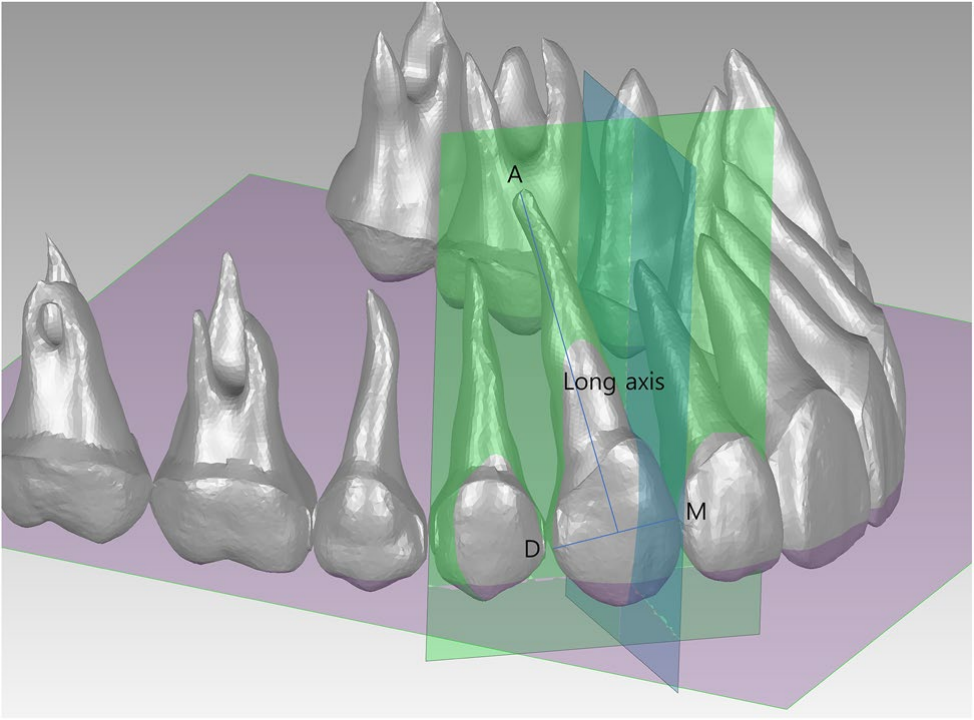
Reference points and planes for angular measurements. Each tooth has a distinct reference plane for angular measurement. The measurement plane for the upper right canine is illustrated as an example. M, mesial point. D, distal point. A, apex point. Long axis, the line segment connecting the A point and the midpoint of the M and D points. Purple, Occlusal plane. It is a plane defined by the distobuccal cusp tips of the maxillary second molars and the contact point of the maxillary central incisors. Green, a reference plane for angulation measurement. It passes through the M and D points of each tooth and is perpendicular to the occlusal plane. Blue, a reference plane for inclination measurement perpendicular to the green plane and occlusal plane.

To define the tooth axes, three reference points were embedded on individual composite teeth in terms of the point features of the Geomagic Control program. The reference points were as follows: the most mesial point (M point), most distal point (D point), and apex point (A point; the apex of single-rooted teeth or the center of furcation of multi-rooted teeth) (Fig. 3).

### Evaluation of root parallelism

For angulation and inclination measurement of each tooth in the three setups, the long axis of each tooth was defined as the line segment connecting the midpoint of the M and D points with the A point. The occlusal plane was defined as the plane passing through the distobuccal cusps of the maxillary left and right second molars and the contact point of the maxillary central incisors in the setup model. Angulation was defined as the angle between the line perpendicular to the occlusal plane and the long axis of each tooth projected to a plane passing through the M and D points of each tooth and perpendicular to the occlusal plane. If A point was distal to the midpoint of the M and D points, the measurement was positive; otherwise, it was negative. Inclination was defined as the angle between the line perpendicular to the occlusal plane and the long axis of each tooth projected to the plane perpendicular to the occlusal plane and to the reference plane for angulation measurement (Fig. 3). If A point was lingual/palatal to the midpoint of the M and D points, the measurement was positive; otherwise, it was negative. Root parallelism was evaluated by calculating the difference in angulation values between the adjacent teeth. In all three setups, the occlusal plane of root setup-2 was commonly used as a reference plane to ensure consistency in angular measurements.

### Measurement of root exposure

**Figure 4:**
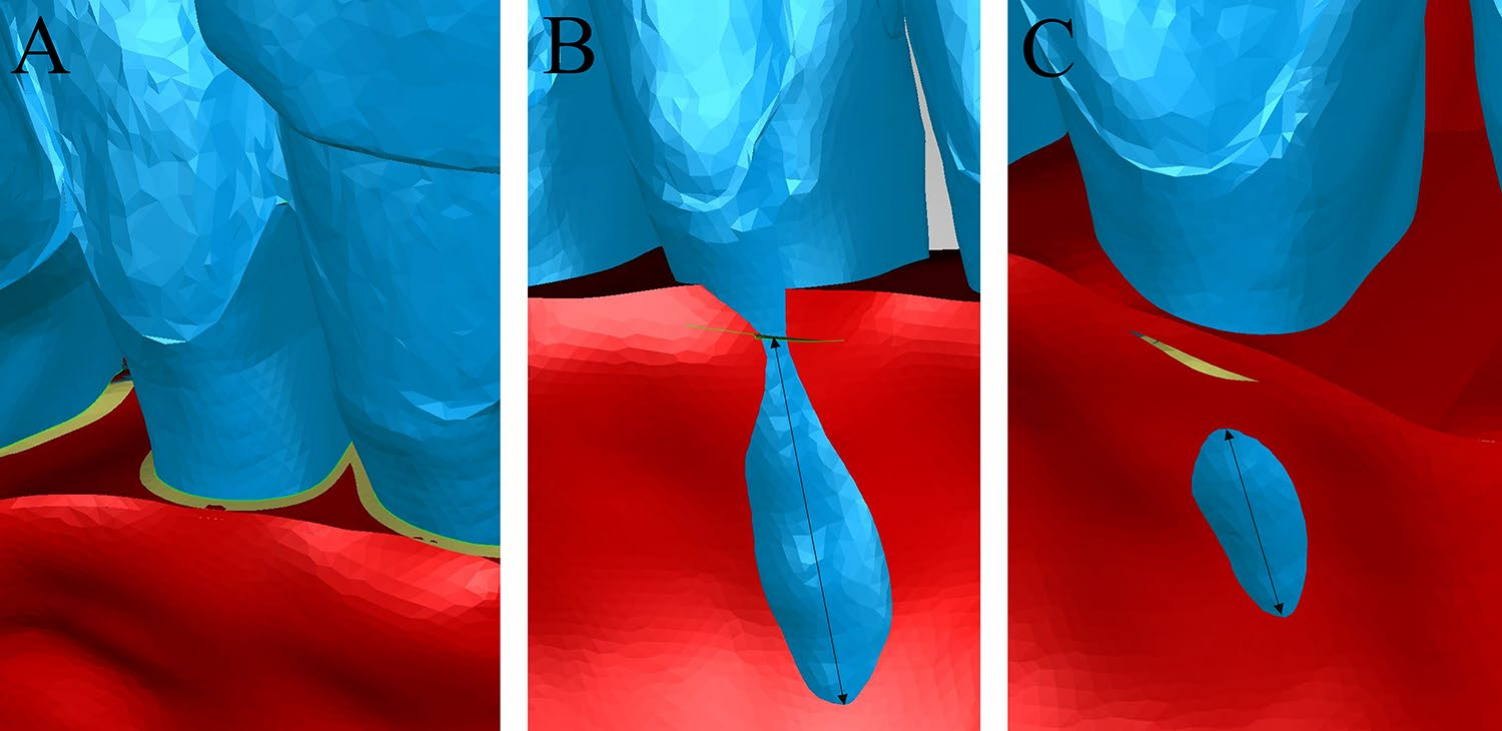
The measurements of root exposure: (**A**), initial state before the setup. The tooth portion above the alveolar crest has been removed from the bone and disc-shaped reference planes indicating the junction of the alveolar bone and roots have been embedded into each composite tooth (yellow circular band). (**B**) The measurement of dehiscence. (**C**), The measurement of fenestration.

In the initial teeth position, a disc-shaped reference plane was placed on each composite tooth with embeded M, D and A reference points. This reference plane was positioned to align with the trimmed surface of the maxilla and mandible and served as a reference for measuring root exposure. The set of composite teeth with disc-shaped reference planes and three reference points was duplicated, and each set was aligned to the three setup models, ensuring the consistency of the reference points for all subsequent measurements. During the process of alveolar bone preparation, the trimmed area where the portion of the tooth above the alveolar crest was separated was covered with a plane resulting in a sharp border. If the root extended beyond this border, the amount of exposure was measured. However, to minimize overestimation of root exposure, we only included teeth with dehiscence or fenestration exceeding 2 mm when comparing the frequency of root exposure between the buccal and palatal/lingual sides^55^. In each setup, the extents of dehiscence and fenestration were measured for each tooth on the buccal and lingual sides. When both dehiscence and fenestration were present, the lengths of each were measured separately and then combined. Dehiscence was measured from the disc-shaped reference planes embedded on each composite tooth (Fig. 4). Additional reference points were embedded on the composite teeth which showed root exposure for linear measurements on the uppermost and lowermost points of dehiscence and/or fenestration.

Throughout this study, we utilized a spreadsheet program (Microsoft Excel 2010; Microsoft, Redmond, WA, USA) for calculation of the linear and angular variables. Instead of using the measurement tool provided by the 3-D analysis program, we employed the 3-D coordinates of the embedded reference points from the Geomagic Control program in each tooth to mitigate potential measurement errors. The linear distances between the two points were directly calculated using their respective coordinates, while the angular values were determined using trigonometric functions. To achieve this, formulas were created to manipulate the coordinates of each tooth's reference points and the reference points forming the occlusal plane, either by horizontal movement or rotation around the coordinate axes.

### Evaluation of occlusion

We measured and compared the ABO-OGS scores by printing rapid prototyping models of the three setups at a layer thickness of 50 μm using a stereolithography apparatus-type 3-D printer (Sindoh A1 + ; Sindoh, Seoul, Korea). The printing material was S-100 M (Graphy, Seoul, Korea). Following curing for 60 s with the MP300 curing machine (Veltz 3D, Incheon, Korea), the models were rinsed with 99% isopropyl alcohol for 5 min.

### Statistical analysis

The intraclass correlation coefficient (ICC) was calculated by repeating the angulation measurement of the teeth in the initial scan and three setups after resetting the M, D, and A points at an interval of 4 weeks in four randomly selected cases. We performed a repeated-measures ANOVA to compare the angular measurements and ABO-OGS scores among the initial scan and three setups. The absolute values were used in the evaluation of angular differences. For each ANOVA test, we performed Mauchly’s Test of Sphericity to assess the sphericity assumption. Post-hoc Bonferroni corrections were used for multiple comparisons. The comparison of the amount of root exposure was conducted using the Friedman test and post-hoc tests were performed with the Wilcoxon signed rank test. We performed the McNemar test to compare the frequency of root exposure between the buccal and palatal sides. Statistical significance was set at *p* < 0.05. We used SPSS software (version 24.0; IBM, Armonk, NY, USA) for all statistical analyses.

### Supplementary Information

Supplementary Table S1.

Supplementary Table S2.

Supplementary Table S3.

## Data Availability

All of the data supporting this work will be made available from the corresponding author upon reasonable request.

## References

[1] Andrews LF (1972). The six keys to normal occlusion. Am. J. Orthod..

[2] Turp JC, Greene CS, Strub JR (2008). Dental occlusion: A critical reflection on past, present and future concepts. J. Oral Rehabil..

[3] Lee SM, Lee JW (2016). Computerized occlusal analysis: correlation with occlusal indexes to assess the outcome of orthodontic treatment or the severity of malocculusion. Korean J. Orthod..

[4] Chiqueto K (2011). Influence of root parallelism on the stability of extraction-site closures. Am. J. Orthod. Dentofac. Orthop..

[5] Wehrbein H, Bauer W, Diedrich P (1996). Mandibular incisors, alveolar bone, and symphysis after orthodontic treatment. A retrospective study. Am. J. Orthod. Dentofac. Orthop..

[6] Bouwens DG, Cevidanes L, Ludlow JB, Phillips C (2011). Comparison of mesiodistal root angulation with posttreatment panoramic radiographs and cone-beam computed tomography. Am. J. Orthod. Dentofac. Orthop..

[7] Pittayapat P (2014). Agreement between cone beam computed tomography images and panoramic radiographs for initial orthodontic evaluation. Oral Surg. Oral Med. Oral Pathol. Oral Radiol..

[8] Hou D, Capote R, Bayirli B, Chan DCN, Huang G (2020). The effect of digital diagnostic setups on orthodontic treatment planning. Am. J. Orthod. Dentofac. Orthop..

[9] Im J, Cha JY, Lee KJ, Yu HS, Hwang CJ (2014). Comparison of virtual and manual tooth setups with digital and plaster models in extraction cases. Am. J. Orthod. Dentofac. Orthop..

[10] Barreto MS, Faber J, Vogel CJ, Araujo TM (2016). Reliability of digital orthodontic setups. Angle Orthod..

[11] Yoon JH (2018). Model analysis of digital models in moderate to severe crowding: In vivo validation and clinical application. Biomed. Res. Int..

[12] Im J (2022). Accuracy and efficiency of automatic tooth segmentation in digital dental models using deep learning. Sci. Rep..

[13] Lee RJ (2015). Three-dimensional monitoring of root movement during orthodontic treatment. Am. J. Orthod. Dentofac. Orthop..

[14] Lee RJ (2018). Three-dimensional evaluation of root position at the reset appointment without radiographs: A proof-of-concept study. Prog. Orthod..

[15] Lee RJ (2018). Accuracy and reliability of the expected root position setup methodology to evaluate root position during orthodontic treatment. Am. J. Orthod. Dentofacial Orthop..

[16] Shin S-H, Hyung-Seog Y, Cha J-Y, Kwon J-S, Hwang C-J (2021). Scanning accuracy of bracket features and slot base angle in different bracket materials by four intraoral scanners: An in vitro study. Materials.

[17] Hou Y, Zhao Y, Wang Y, Wang S, Liu Y (2015). A pilot study of root position in orthodontic diagnosis model set-up. Zhonghua Kou Qiang Yi Xue Za Zhi.

[18] Mavropoulos A, Karamouzos A, Kiliaridis S, Papadopoulos MA (2005). Efficiency of noncompliance simultaneous first and second upper molar distalization: A three-dimensional tooth movement analysis. Angle Orthod..

[19] Mohamed RN, Basha S, Al-Thomali Y (2018). Maxillary molar distalization with miniscrew-supported appliances in Class II malocclusion: A systematic review. Angle Orthod..

[20] Pham V, Lagravère MO (2017). Alveolar bone level changes in maxillary expansion treatments assessed through CBCT. Int. Orthod..

[21] Nakada T, Motoyoshi M, Horinuki E, Shimizu N (2016). Cone-beam computed tomography evaluation of the association of cortical plate proximity and apical root resorption after orthodontic treatment. J. Oral Sci..

[22] Vardimon AD, Oren E, Ben-Bassat Y (1998). Cortical bone remodeling/tooth movement ratio during maxillary incisor retraction with tip versus torque movements. Am. J. Orthod. Dentofac. Orthop..

[23] Park G (2020). Posterior anatomic limit for distalization of maxillary dentition.

[24] Kim SJ, Choi TH, Baik HS, Park YC, Lee KJ (2014). Mandibular posterior anatomic limit for molar distalization. Am. J. Orthod. Dentofac. Orthop..

[25] Garib DG, Henriques JF, Janson G, de Freitas MR, Fernandes AY (2006). Periodontal effects of rapid maxillary expansion with tooth-tissue-borne and tooth-borne expanders: A computed tomography evaluation. Am. J. Orthod. Dentofac. Orthop..

[26] Wang H, Zhao N, Li P, Shen G (2019). A cone-beam computed tomography analysis of angulation and inclination of whole tooth and clinical crown in adults with normal occlusion. Orthod. Craniofac. Res..

[27] Vermylen K, De Quincey GNT, van Hof MA, Wolffe GN, Renggli HH (2005). Classification, reproducibility and prevalence of root proximity in periodontal patients. J. Clin. Periodontol..

[28] Graber TM (1966). Postmortems in posttreatment adjustment. Am. J. Orthod..

[29] Edwards JG (1971). The prevention of relapse in extraction cases. Am. J. Orthod..

[30] Hatasaka HH (1976). A radiographic study of roots in extraction sites. Angle Orthod..

[31] Yeom HG (2020). Correlation between spatial resolution and ball distortion rate of panoramic radiography. BMC Med. Imaging.

[32] Lucchesi MV, Wood RE, Nortje CJ (1988). Suitability of the panoramic radiograph for assessment of mesiodistal angulation of teeth in the buccal segments of the mandible. Am. J. Orthod. Dentofac. Orthop..

[33] Garcia-Figueroa MA, Raboud DW, Lam EW, Heo G, Major PW (2008). Effect of buccolingual root angulation on the mesiodistal angulation shown on panoramic radiographs. Am. J. Orthod. Dentofac. Orthop..

[34] Tong H, Enciso R, Van Elslande D, Major PW, Sameshima GT (2012). A new method to measure mesiodistal angulation and faciolingual inclination of each whole tooth with volumetric cone-beam computed tomography images. Am. J. Orthod. Dentofac. Orthop..

[35] Lee WC (2016). Crown morphology of the mandibular first molars with distolingual roots. J. Dent. Sci..

[36] Casko JS (1998). Objective grading system for dental casts and panoramic radiographs. American Board of Orthodontics. Am. J. Orthod. Dentofac. Orthop..

[37] Handelman CS (1996). The anterior alveolus: Its importance in limiting orthodontic treatment and its influence on the occurrence of iatrogenic sequelae. Angle Orthod..

[38] Evangelista K (2010). Dehiscence and fenestration in patients with Class I and Class II Division 1 malocclusion assessed with cone-beam computed tomography. Am. J. Orthodont. Dentofac. Orthoped..

[39] Leung CC, Palomo L, Griffith R, Hans MG (2010). Accuracy and reliability of cone-beam computed tomography for measuring alveolar bone height and detecting bony dehiscences and fenestrations. Am. J. Orthod. Dentofac. Orthop..

[40] Rupprecht RD, Horning GM, Nicoll BK, Cohen ME (2001). Prevalence of dehiscences and fenestrations in modern American skulls. J. Periodontol..

[41] Jorgic-Srdjak K, Plancak D, Bosnjak A, Azinovic Z (1998). Incidence and distribution of dehiscences and fenestrations on human skulls. Coll. Antropol..

[42] Davies RM, Downer MC, Hull PS, Lennon MA (1974). Alveolar defects in human skulls. J. Clin. Periodontol..

[43] Edel A (1981). Alveolar bone fenestrations and dehiscences in dry Bedouin jaws. J. Clin. Periodontol..

[44] Ye N (2012). Accuracy of in-vitro tooth volumetric measurements from cone-beam computed tomography. Am. J. Orthod. Dentofac. Orthop..

[45] Liu Y (2010). The validity of in vivo tooth volume determinations from cone-beam computed tomography. Angle Orthod..

[46] Dong T (2019). Accuracy of in vitro mandibular volumetric measurements from CBCT of different voxel sizes with different segmentation threshold settings. BMC Oral Health.

[47] Hassan B, Couto Souza P, Jacobs R, de Azambuja Berti S, van der Stelt P (2010). Influence of scanning and reconstruction parameters on quality of three-dimensional surface models of the dental arches from cone beam computed tomography. Clin. Oral Investig..

[48] Fourie Z, Damstra J, Schepers RH, Gerrits PO, Ren Y (2012). Segmentation process significantly influences the accuracy of 3D surface models derived from cone beam computed tomography. Eur. J. Radiol..

[49] Sathapana S, Forrest A, Monsour P, Naser-ud-Din S (2013). Age-related changes in maxillary and mandibular cortical bone thickness in relation to temporary anchorage device placement. Aust. Dent. J..

[50] Cao T, Xu L, Shi J, Zhou Y (2015). Combined orthodontic-periodontal treatment in periodontal patients with anteriorly displaced incisors. Am. J. Orthod. Dentofac. Orthop..

[51] Ramos AL, Dos Santos MC, de Almeida MR, Mir CF (2020). Bone dehiscence formation during orthodontic tooth movement through atrophic alveolar ridges. Angle Orthod..

[52] Yagci A (2012). Dehiscence and fenestration in skeletal Class I, II, and III malocclusions assessed with cone-beam computed tomography. Angle Orthod..

[53] Lee RJ (2019). Accuracy of the expected root position setup to monitor root angulations and inclinations during orthodontic treatment: A pilot study. J. Indian Orthod. Soc..

[54] Yushkevich PA (2006). User-guided 3D active contour segmentation of anatomical structures: Significantly improved efficiency and reliability. Neuroimage.

[55] Sun L (2019). Changes of alveolar bone dehiscence and fenestration after augmented corticotomy-assisted orthodontic treatment: a CBCT evaluation. Prog. Orthod..

