# Supplementary material for: Three-dimensional evaluation of a virtual setup considering the roots and alveolar bone in molar distalization cases
Source: Sci Rep. 2023 Sep 11;13:14955. doi: 10.1038/s41598-023-41480-z (PMC10495328; doi:10.1038/s41598-023-41480-z)
Supplement: Supplementary file 1 — Supplementary Table S1. [file 41598_2023_41480_MOESM1_ESM.docx]

**Supplementary Table S1**. Comparison of buccolingual inclination of teeth axes among the three setup models

|  | Tooth | Crown setup^a^ | | Root setup-1^b^ | | Root setup-2^c^ | | *p*-value |  | Post hoc |
| --- | --- | --- | --- | --- | --- | --- | --- | --- | --- | --- |
|  | type | Mean | SD | Mean | SD | Mean | SD |  |  |  |
| Maxillary | Incisor | 32.64 | 5.45 | 32.39 | 5.20 | 31.56 | 5.61 | 0.051 |  | NA |
|  | Canine | 19.28 | 3.77 | 19.46 | 3.67 | 19.63 | 4.27 | 0.648 |  | NA |
|  | Premolar | 10.92 | 4.33 | 10.79 | 4.29 | 11.04 | 4.38 | 0.359 |  | NA |
|  | Molar | 10.81 | 4.73 | 10.83 | 4.71 | 12.22 | 5.34 | 0.000 | * | a>c b>c |
| Mandibular | Incisor | 18.80 | 5.14 | 18.59 | 4.89 | 17.27 | 5.93 | 0.007 | * | a>c b>c |
|  | Canine | 15.76 | 4.26 | 15.59 | 3.88 | 13.85 | 5.71 | 0.001 | * | a>c b>c |
|  | Premolar | 7.93 | 4.70 | 6.57 | 3.84 | 5.34 | 3.94 | 0.000 | * | a>b a>c b>c |
|  | Molar | -3.51 | 5.18 | -3.65 | 5.34 | -4.62 | 5.89 | 0.003 | * | a>c b>c |

*p*-values were derived from repeated measure ANOVA, **p* < 0.05.

Bonferroni adjustment was used for multiple comparisons.

If A point was lingual/palatal to the midpoint of the M and D points, the measurement was positive; otherwise, it was negative.

NA = not applicable.
