# Supplementary material for: Three-dimensional evaluation of a virtual setup considering the roots and alveolar bone in molar distalization cases
Source: Sci Rep. 2023 Sep 11;13:14955. doi: 10.1038/s41598-023-41480-z (PMC10495328; doi:10.1038/s41598-023-41480-z)
Supplement: Supplementary file 2 — Supplementary Table S2. [file 41598_2023_41480_MOESM2_ESM.docx]

**Supplementary Table S2**. Movement of the central incisors and first molars during the three setup methods

|  | Tooth type | Crown setup^a^ | | Root setup-1^b^ | | Root setup-2^c^ | | *p*-value | Post hoc |
| --- | --- | --- | --- | --- | --- | --- | --- | --- | --- |
|  |  | Mean | SD | Mean | SD | Mean | SD |  |  |
| Maxillary | Central incisors | 1.82 | 1.00 | 1.82 | 1.00 | 1.72 | 1.01 | 0.097 | NA |
|  | First molars | 1.92 | 1.39 | 1.90 | 1.38 | 1.87 | 1.39 | 0.274 | NA |
| Mandibular | Central incisors | 0.85 | 1.05 | 0.85 | 1.06 | 0.79 | 0.94 | 0.292 | NA |
|  | First molars | 1.82 | 1.01 | 1.81 | 1.01 | 1.79 | 1.04 | 0.609 | NA |

*p*-values were derived from repeated measure ANOVA, **p* < 0.05.

Bonferroni adjustment was used for multiple comparisons.

NA = not applicable.
