# Supplementary material for: Three-dimensional evaluation of a virtual setup considering the roots and alveolar bone in molar distalization cases
Source: Sci Rep. 2023 Sep 11;13:14955. doi: 10.1038/s41598-023-41480-z (PMC10495328; doi:10.1038/s41598-023-41480-z)
Supplement: Supplementary file 3 — Supplementary Table S3. [file 41598_2023_41480_MOESM3_ESM.docx]

**Supplementary Table S3**. Cephalometric descriptive statistics of the enrolled patients

|  |  | | | Mean | SD |
| --- | --- | --- | --- | --- | --- |
| ANB difference (°) | | | | 2.11 | 2.41 |
| APDI (°) |  | | | 84.41 | 6.25 |
| Wits appraisal (mm) | | | | -3.02 | 3.09 |
| Mandibular body length (mm) | | | | 79.94 | 5.78 |
| SN to Go-Me (°) | | | | 34.69 | 5.51 |
| U1 to SN (°) |  | | | 105.29 | 8.19 |
| IMPA (°) |  | | | 96.52 | 8.36 |
| Upper lip to E-plane (mm) | | |  | 1.31 | 2.65 |
| Lower lip to E-plnae (mm) | | |  | 2.00 | 3.78 |
| Overjet (mm) |  | | | 2.94 | 1.47 |
| Overbite (mm) | |  | | 2.26 | 1.33 |
